# Distinct miRNA profiles in human amniotic tissue and its vesicular and non-vesicular secretome

**DOI:** 10.3389/fcell.2025.1692501

**Published:** 2025-10-29

**Authors:** Nefertiti Chaves-Solano, Silvio Kau-Strebinger, Johannes Oesterreicher, Marianne Pultar, Wolfgang Holnthoner, Johannes Grillari, Simone Hennerbichler, Andreas Brandstetter, Andreas Spittler, Matthias Hackl, Susanne Wolbank, Asmita Banerjee, Adelheid Weidinger

**Affiliations:** ^1^ Ludwig Boltzmann Institute for Traumatology, The Research Center in Cooperation with AUVA, Vienna, Austria; ^2^ Austrian Cluster for Tissue Regeneration, Vienna, Austria; ^3^ Department of Biomedical Sciences and Pathobiology, Centre of Pathobiology, Unit of Morphology, University of Veterinary Medicine, Vienna, Austria; ^4^ TAmiRNA GmbH, Vienna, Austria; ^5^ Institute for Molecular Biotechnology, University of Natural Resources and Life Sciences, Vienna, Austria; ^6^ Red Cross Blood Transfusion Service of Upper Austria, Linz, Austria; ^7^ Department of Gynecology and Obstetrics, St. Josef Krankenhaus GmbH Vienna, Vienna, Austria; ^8^ Core Facilities, Unit Flow Cytometry and Surgical Research Laboratories, Medical University of Vienna, Vienna, Austria

**Keywords:** human amniotic membrane, perinatal tissue, miRNA, extracellular vesicles, vesicular secretome, non-vesicular secretome, tissue regeneration

## Abstract

**Introduction:**

The human amniotic membrane (hAM) has largely been used in tissue regeneration and wound healing applications. A promising alternative to decellularized hAM or isolated cells is the usage of native viable hAM which contains and releases cell-derived bioactive factors that are known to enhance tissue regeneration. MicroRNAs (miRNAs) are known regulators of gene expression at post-transcriptional level and are important drivers of regeneration processes in several tissues. In this study, we characterized the miRNA profile of hAM tissue and its vesicular and non-vesicular secretome in the reflected and placental hAM as two spatially and physiologically distinct regions.

**Methods:**

Extracellular vesicles were enriched from the secretome by size exclusion chromatography (SEC). Small RNAs were determined by Next Generation Sequencing in the conditioned medium and in tissue.

**Results:**

After SEC, we identified predominantly small hAM-derived EVs (≤200 nm) expressing CD81. The highest percentage of miRNA relative to all mapped reads was found in tissue (15%–40%), while 2%–15% were protein-bound and 3%–6% associated with EVs. Unsupervised clustering revealed distinct clusters of miRNA expression according to sample fraction (EV-associated, protein-bound, and tissue) and amniotic regions (reflected, placental). Gene ontology analysis linked EV-associated and tissue miRNAs to (smooth) muscle proliferation, while protein-bound miRNAs were associated with connective tissue development, chondrocyte differentiation and glial cell proliferation. Furthermore, correlation analysis of tissue miRNAs and extracellular expression identified EV-associated and protein-bound miRNAs specifically released from the tissue.

**Conclusion:**

These findings support the assumption that native viable hAM could serve as a miRNA source for applications in regenerative medicine.

## 1 Introduction

The human amniotic membrane (hAM), a tissue of embryonic origin, has served as transplant material and source for cells in wound healing and tissue regeneration applications for more than 100 years (Silini et al., 2015). Over the course of history, the hAM has been utilized in various forms, which mostly included decellularized, denuded, cryo-conserved, freeze-dried or powdered preparations (Silini et al., 2015). The bioactive factors that support tissue regeneration during clinical applications can originate from different components within the hAM. On the one hand, the extracellular matrix layers of the hAM retain growth factors, hormones (Comperat et al., 2024) and structural molecules such as collagens (Gunasekaran et al., 2020), fibronectin (Lockwood et al., 1991), and hyaluronic acid (Zhu et al., 2020) even after cell removal. On the other hand, vital (non-decellularized or non-denuded) hAM contains two cell types with stem cell characteristics, human amniotic membrane epithelial cells (hAECs) and human amniotic membrane mesenchymal stromal cells (hAMSCs) (Portmann-Lanz et al., 2006; Miki et al., 2007). Besides their demonstrated differentiation capacity towards all three germ layers *in vitro* and *in vivo* (Sakuragawa et al., 1996; Kakishita et al., 2003; In’t Anker et al., 2004; Portmann-Lanz et al., 2006), hAM cells also release a variety of bioactive factors, such as nerve growth factor (NGF) (Zhang et al., 2019), brain-derived neurotrophic factor (BDNF) (Banerjee et al., 2014), glial cell-derived neurotrophic factor (GDNF) (Banerjee et al., 2014), angiogenic factors (Wolbank et al., 2009; Duan-Arnold et al., 2015) and epidermal growth factor (EGF) (Gicquel et al., 2009) that are all known to enhance tissue regeneration.

MicroRNAs (miRNAs), a class of small non-coding RNAs, are regulators of gene expression at post-transcriptional level by base-pairing with the 3′UTR of the target mRNA, thereby inhibiting (O’Brien et al., 2018) or activating translation (Vasudevan and Steitz, 2007). Importantly, miRNAs are involved in many cellular processes such as cell proliferation (Rodrigues et al., 2018) and differentiation (Berardi et al., 2012), apoptosis (Su et al., 2015), or in maintaining cellular homeostasis (van Wijk et al., 2022). Regarding their function, it is assumed that miRNAs act in an autocrine manner or are secreted into the extracellular environment participating in cell-cell communication (Makarova et al., 2016). Intriguingly, miRNAs are not only involved in cell communication within a species, but they also seem to engage in cross-kingdom interactions (Zhang et al., 2012). Depending on their biological function, secreted miRNAs can be packed into extracellular vesicles (EVs, “EV-associated miRNA”) (Valadi et al., 2007; Zernecke et al., 2009) or bound to proteins (“protein-bound miRNA”) (Arroyo et al., 2011). EV-associated miRNAs are protected by the surrounding bi-lipid membrane (Valadi et al., 2007), protein-bound miRNAs are stabilized by proteins including Argonaut 2 (AGO2) complexes (Arroyo et al., 2011; Turchinovich and Burwinkel, 2012; Geekiyanage et al., 2020), nucleophosmin (NPM1) (Wang et al., 2010), and/or lipoproteins (Vickers et al., 2011). However, non-vesicle-associated miRNAs are possibly more prone to degradation (Köberle et al., 2013), suggesting that their biological function may depend on their packaging. In cellular lysates lacking Ago expression, it has been shown that mature single-stranded miRNAs are unstable and rapidly degrade by endogenous nucleases, whereas in Ago-expressing lysates, miRNAs are significantly more stable (Park et al., 2017). In any case, circulating miRNAs are of high potential as biomarkers in various clinical settings including tissue regeneration (Grillari et al., 2021).

While several studies have investigated proteins such as growth factors as the main bioactive factors released from hAM, only few studies have reported on the miRNA composition of the hAM secretome. For example, it has been reported that miRNAs of chorioamniotic membrane (Montenegro et al., 2009) and hAM (Kim et al., 2011; Son et al., 2019) impact the post-transcriptional regulation of gene expression during human parturition. Ragni and colleagues isolated hAMSCs and characterized their secreted EV-associated miRNAs (Ragni et al., 2020; 2021b; 2021a). Also, miRNA expression in the exosomal fraction of the conditioned medium of amniotic fluid stem cells was analyzed (Castelli et al., 2021).

**FIGURE 1:**
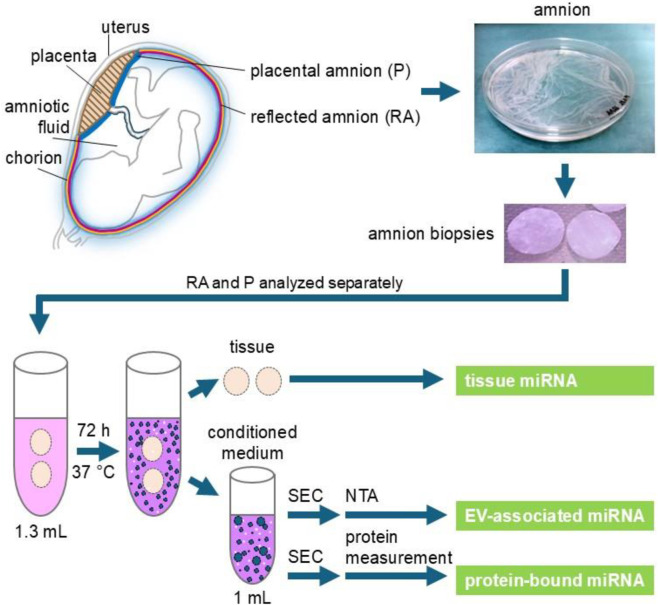
Experimental scheme. Human placentae were collected after cesarean sections. Reflected amnion (RA) and placental amnion (P) were separately peeled off the placenta and biopsy punches of 26 mm diameter were prepared. For 1 mL supernatant (conditioned medium), two biopsies each of reflected and placental amnion were incubated in 1.3 mL of serum-free culture medium for 72 h at 37 °C. The supernatant was subjected to size exclusion chromatography (SEC) to enrich extracellular vesicles (EV). Nanoparticle tracking analysis (NTA) was performed to measure EV quantity in RA- and P-EV preparations. Protein content was measured to separate the EV fraction from the protein fraction. MiRNA in tissue, protein (non-vesicular) fraction and EV fraction was analyzed by next-generation sequencing.

Considering the indispensable role of miRNAs in regenerative processes, comprehensive insight into the miRNA profile of the hAM tissue and its secretory derivatives is crucial for elucidating the mechanisms behind its potential. In this study, we analyzed the miRNA profile of hAM tissue samples (tissue miRNAs) and their corresponding vesicular and non-vesicular secretome (secreted miRNAs) using an *ex vivo* approach (Figure 1). As there is evidence that different regions of the amniotic membrane may have different regenerative potentials (Weidinger et al., 2021), we separately analyzed samples of the placental and reflected region of the hAM.

## 2 Materials and methods

### 2.1 Preparation of human amniotic membrane

Human placentae were collected after cesarean sections with informed consent and approval from the local Ethics committee (Ethikkommission des Landes Oberösterreich, no. 200, Ethikkommission der Wiener Krankenhäuser der Vinzenz Gruppe, Ethics committee no. 1011/2022). Preparation of the hAM was performed as described previously (Poženel et al., 2019). Briefly, reflected amnion (RA) and placental amnion (P) were separately peeled off the placenta and washed thoroughly with phosphate buffered saline (1X PBS, Sigma-Aldrich, Austria).

### 2.2 Preparation of hAM conditioned medium

For 1 mL supernatant (conditioned medium), we prepared two biopsy punches of 26 mm diameter each from RA and P and incubated them in 1.3 mL of serum-free cell culture medium (Dulbecco Modified Eagle’s medium/high glucose (Sigma-Aldrich, Austria) supplemented with 1% v/v L-glutamine (Sigma-Aldrich, Austria), and 1% v/v penicillin/streptomycin (Sigma-Aldrich, Austria)) at 37 °C, in a humidified atmosphere with 5% CO_2_. After 72 h, supernatants (conditioned media) were collected. Differential centrifugation was performed at 1,500 × g for 10 min, followed by a second centrifugation at 10,000 × g for 30 min. The supernatants were stored at – 80 °C until further processing.

### 2.3 Enrichment of EVs by size exclusion chromatography (SEC) for characterization of sample preparation

1 mL of supernatant (conditioned medium) of each hAM region was concentrated to approximately 150 µL using Amicon 100 kDa MWCO ultrafiltration units (Merck Millipore, United States) and further subjected to SEC using qEVsingle 70 nm columns (Izon Science, New Zealand, 15928090) according to the manufacturer´s specifications. In brief, 150 µL concentrated conditioned medium was loaded onto SEC columns pre-equilibrated with freshly filtered (filter pore size 0.22 µm) PBS (Sigma-Aldrich, United States, D8537).

After automatically discarding the void volume, elution fractions were harvested using PBS as the elution buffer. A total of 16 fractions (each 200 µL) were collected and analyzed for concentration and size distribution of nanoparticles, and protein concentration using nanoparticle tracking analysis (NTA) and bicinchoninic acid assay, respectively. Based on particle quantity, SEC fractions 1 - 8 were analyzed for EV marker expression by fluorescence-triggered flow cytometry (FT-FC), transmission electron microscopy (TEM) and Western blot, following MISEV2023 guidelines to the extent feasible (Welsh et al., 2024).

### 2.4 Nanoparticle tracking analysis (NTA)

Nanoparticle tracking analysis (NTA) was performed to characterize nanoparticle size and quantity in RA- and P-EV preparations using a ZetaView 430 QUATT device (Particle Metrix, Germany). Samples of 3 donors (biological replicates) were analyzed at a constant temperature of 25 °C. The device settings were configured with a shutter speed of 100, sensitivity set to 75, and the measurement mode set to 1 cycle and 11 positions. Quality control parameters adhered to the manufacturer’s recommendations, ensuring that the average counted particles per frame ranged between 100 and 400, and the number of traced particles exceeded 500. Data were acquired and exported as. txt files using ZetaView software version 8.05.12 (Particle Metrix GmbH, Germany). The exported data was analyzed with MS Excel software (Microsoft Inc., United States) and visualized in GraphPad Prism version 9 (GraphPad, United States).

### 2.5 Fluorescence-triggered flow cytometry analysis (FT-FC)

FT-FC was performed as described previously (Oesterreicher et al., 2020). 50 μL of enriched EV fractions were mixed with 50 µL of CellMask Green (CMG) (Invitrogen, United States, C37608), diluted 1:2000 with ddH_2_O, and incubated for 30 min at 37 °C. The staining of the respective antigens was performed by subsequent addition of 4 μL of 1:100 predilute PE-labelled antibodies for CD9 (Miltenyi Biotech, 130-118-865), CD63 (Miltenyi Biotech, Germany, 130-118-077), CD81 (Miltenyi Biotech, Germany, 130-118-481), and isotype IgG1 (Miltenyi Biotech, Germany, 130-113-438). Samples were incubated in the dark for 30 min. Controls included PBS instead of sample, and temperature control was performed by incubating the samples for 10 min at 95 °C. Measurements were taken using a CytoFlexS flow cytometer and CytExpert software version 1.2 (Beckman Coulter GmbH, Brea, CA, United States). Data analysis was performed using FlowJo software version 10 (FlowJo LLC, United States).

### 2.6 Micro bicinchoninic acid (BCA) protein assay

To differentiate EV-enriched SEC fractions from free protein-enriched SEC fractions in our preparations, the Micro BCA™ Protein Assay Kit (Thermo Fisher Scientific, #23235, United States) was performed following the manufacturer’s instructions. Briefly, 150 µL of each standard and 150 µL SEC-fraction samples (1:3 diluted) was pipetted into a microplate well (product no. 15041) and 150 µL of the working reagent was added to each well. After mixing on a shaker for 30 s, the covered plate was incubated at 37 °C for 2 h. Absorbance was measured at 562 nm using a microplate reader (BMG Labtech, Polarstar Omega, Germany) and values were interpolated to calculate the BSA standard concentration versus sample concentration in µg/mL using GraphPad Prim V.9 (GraphPad, United States).

### 2.7 Western blot

To increase the amount and concentration of sample required for qualitative Western blot analysis, EVs were enriched by differential ultracentrifugation. The RA and P tissue were incubated to end up in 45 mL medium each. After 72 h, supernatants were collected and differentially centrifuged at 1,500 × g for 10 min, followed by a second centrifugation at 10,000 × g for 30 min. After that, the supernatants were transferred into ultracentrifugation tubes (40PC tube, Eppendorf, Japan) and centrifuged in an ultracentrifuge CP100NX (Eppendorf, Japan) using a swing bucket rotor (P32ST, Eppendorf, Japan) at 1,000,00 × g for 2 h (excluding acceleration time) at 4 °C under vacuum. The pellets were resuspended in sterile-filtered 1X PBS and stored at −80 °C until further use.

Western blot analysis was performed on EV preparations from RA and P, as well as corresponding RA and P tissue controls from the same donors (matched EV-tissue pairs, n = 3). Samples were lysed using RIPA buffer, and protein concentrations were determined using the DC Protein Assay Kit II (BioRad, United States, #5000112). 5 μg of protein per lane was loaded for both EV and tissue samples. Samples were heated to 95 °C for 8 min, and proteins were separated on a 10% acrylamide gel. All samples were run under reducing conditions, except for CD63 and CD81, which were run under non-reducing conditions. Electrophoresis was conducted at a constant current of 200 V for 50 min. Proteins were then transferred onto nitrocellulose membranes (BioRad, United States, #1620115, 0.45 µm) at 350 mA for 60 min. Membranes were blocked with a commercial Western blocking reagent (Roche, Switzerland, #11921673001) in 1X TBST (1:10) for 2 h. Primary antibodies were incubated on a roller at 4 °C overnight in blocking solution: a-CD63 mAb (Invitrogen, United States, clone Ts63, #10628D, 1:500), a-CD81 mAb (BD Transduction Laboratories, United States, clone JS-81, #555675, 1:1,000), a-LAMP1 mAb (Cell Signaling, United States, clone C54H11, #3243, 1:1,000), a-TSG101 mAb (BD Transduction Laboratories, United States, clone 51/TSG101, #612696, 1:250), a-Flot-1 mAb (Cell Signaling, United States, clone D2V7J, #18634, 1:1,000), a-HSP70 mAb (Antikörper-online, Germany, clone C92F3A-5, #ABIN361708, 1:500), a-beta actin mAb (Santa Cruz, United States, clone C4, #sc-47778, 1:500), and a-calnexin pAb (Sigma Aldrich, United States, #C4731, 1:2000). After washing, membranes were incubated with HRP-conjugated secondary antibodies (a-mouse or a-rabbit IgG, Cytiva, United Kingdom, #NA931 and #NA934, both 1:5,000) for 1 h at RT. Chemiluminescence detection was performed using ECL Western Blotting Detection Reagents (Cytiva, United Kingdom, #RPN2209) and visualized with a BioRad ChemiDoc Station (BioRad, United States).

### 2.8 Transmission electron microscopy (TEM)

#### 2.8.1 Immunogold labeling

TEM was further used to analyze CD81 display of SEC-isolated RA and P amnion EVs from one donor. After SEC, 10 µL of RA and P-EV suspensions (adjusted to contain 1E+07 particles) were placed on 300 mesh hexagonal formvar/carbon-coated Ni grids (EMS, United States, FCF300H-Ni-50) and incubated for 75 min at room temperature (RT). The grids were washed with freshly filtered (0.22 µm) PBS and dried with filter paper. Grids were then incubated for 15 min with freshly filtered (0.22 µm) 0.2% BSA (Roche, Germany, 10735078001) in filtered 1X PBS (w/o) (pH 7.4) to block non-specific binding. Next, the grids were incubated with a mouse anti-human CD81 antibody (clone JS-81; BD Biosciences, United States, 555,675) diluted 1:50 with 0.2% BSA in PBS (pH 7.4) for 40 min at RT. After washing with filtered PBS, grids were incubated with 10 nm colloidal gold-conjugated anti-mouse IgG (Sigma-Aldrich, United States, G7652) diluted 1:50 with 0.2% BSA in filtered PBS (pH 8.0) for 20 min at RT. Unbound antibodies were washed away with filtered PBS. The grids were then fixed with 1% freshly filtered glutaraldehyde (Sigma-Aldrich, United States, G5882) in PBS (pH 7.4) and washed in ddH_2_O. Finally, the grids were stained with 2% freshly filtered aqueous uranyl acetate (Thermo Fisher Scientific, United States, 18-607-644) for 1 min. After air drying overnight, EV samples (CD81 labeled and unlabeled controls) and a buffer background control were examined using a Zeiss EM 900 transmission electron microscope equipped with a slow-scan CCD 2K wide-angle dual-speed camera (TRS, Germany) and ImageSP software version 1.2.11.15 (TRS, Germany and SYSPROG, Belarus).

### 2.9 Analysis of RNA profile

#### 2.9.1 Isolation of EV and protein fraction

1 mL of cell culture supernatants (n = 4 biological replicates) were concentrated to 300 µL using 30 kDa Amicon tubes (Merck Millipore, United States). EVs and protein fractions were isolated with qEV single columns (70 nm, Gen2, Izon, New Zealand) in accordance with the manufacturer’s instructions and concentrated to 300 µL for miRNA analysis.

#### 2.9.2 RNA extraction

##### 2.9.2.1 EV and protein SEC fraction

Total RNA was extracted from 200 μL EV or protein fraction using the miRNeasy Mini Kit (cat. No. 217004, Qiagen, Germany). Samples were homogenized with 1 mL Qiazol and rigorous mixing for 10 s. Then, samples were incubated at room temperature for 5 min. 200 μL chloroform were added, lysates were mixed again, and left at room temperature for 3 min. Afterwards, all samples were centrifuged at 120,00 × g for 15 min at 4 °C. Precisely 650 µL aqueous phase were transferred to fresh tubes, and 7 µL glycogen (5 mg/mL) were added for enhanced precipitation. For binding to RNeasy Mini Spin Columns and washing steps, a QIAcube liquid handling robot was used. RNA was eluted in 30 µL nuclease-free water (RA_tissue_ mean 176.0 ng/μL, P_tissue_ mean 315.8 ng/μL; 100 ng of RNA was used for library preparation) and stored at −80 °C until further analysis.

##### 2.9.2.2 Tissue

Total RNA was extracted from snap-frozen amnion tissue (n = 4) using the miRNeasy Mini Kit (Qiagen, Germany). Each sample was homogenized with 350 µL Qiazol and lysing matrix Z (MP Biomedicals, country) in a Bead Beater FastPrep24 5g (MP Biomedicals, Germany) then 350 µL Qiazol were added. Samples were then incubated at room temperature for 10 min 140 μL chloroform were added to the lysates followed by centrifugation at 120,00x g for 15 min at 4 °C. Precisely 350 µL upper aqueous phase were transferred to fresh tubes. For binding to RNeasy Mini Spin Columns and washing steps, a QIAcube liquid handling robot was used. RNA was eluted in 30 µL nuclease-free water and stored at −80 °C until further analysis. RNA quality and concentration were determined with the RNA 6000 Nano Kit (Agilent, Germany).

#### 2.9.3 Small RNA sequencing

For both the EV and protein fraction, 8.5 µL total RNA were used as input for the generation of small RNA sequencing libraries, while for the tissue, 100 ng total RNA were used. Libraries were generated with the RealSeq Biofluids library preparation kit (RealSeq Biosciences). To each sample, 1 µL miND spike-in standards (TAmiRNA, Austria) were added during the first step. Adapter-ligated libraries were amplified (20 cycles for EV and protein fractions; 19 cycles for tissue) using barcoded Illumina reverse primers in combination with the Illumina forward primer. Library quality control was performed using DNA 1000 chips (Agilent, Germany). All samples were pooled equimolarly and processed with the Blue Pippin system (Sage Science, United States) using 3% agarose size selection cassettes, following the manufacturer’s instructions (size range: 130-160 bp). Sequencing was performed on an Illumina NovaSeq SP SR100 (Illumina, United States).

#### 2.9.4 Data analysis

##### 2.9.4.1 Next-generation sequencing

Next-generation sequencing (NGS) data was analyzed using the miND® analysis pipeline (Diendorfer et al., 2022) and evaluated with fastQC v0.11.9 (Andrews, 2010) and multiQC v1.14 (Ewels et al., 2016). Reads were adapter trimmed and quality filtered using cutadapt v3.3 (Martin, 2011). Mapping steps were performed with Bowtie v1.3.0 (Langmead et al., 2009) and miRDeep2 v2.0.1.2 (Friedländer et al., 2012). Reads were initially mapped against the genomic reference GRCh38. p12 by Ensembl (Zerbino et al., 2018) allowing two mismatches and subsequently against miRBase v22.1 (Griffiths-Jones, 2004), filtered for microRNAs of hsa, allowing one mismatch. For a general RNA composition, non-microRNA mapped reads were mapped against RNAcentral v19.0 (Sweeney et al., 2019) and assigned to RNA species of interest.

Statistical analysis of NGS data was conducted with R V4.0, pheatmap V1.0.12, pcaMethods V1.82 and genefilter V1.72. Differential expression analysis was done with edgeR V3.32 (Robinson et al., 2009) using the quasi-likelihood negative binomial generalized log-linear model functions. The independent filtering method of DESeq2 (Love et al., 2014) was adapted for use with edgeR to remove low abundant microRNAs. Additional NGS quality control and absolute quantification of microRNAs was done using miND® spike-ins (Khamina et al., 2022) based on a linear regression model. For unsupervised exploration and hierarchical clustering, only miRNAs that show an RPM of 5 in at least 33% of the samples were included in the heat map and clustering to increase robustness.

##### 2.9.4.2 Venn diagram

To compare the miRNA from each sample type of the supernatant (EVs from reflected amnion, EVs from placental amnion, proteins from reflected amnion, and proteins from placental amnion) and identify common and unique miRNAs among the top 20 most abundant miRNAs, Venn diagrams were generated using the ggplot2 and ggvenn R packages (R version 4.4.0).

##### 2.9.4.3 Gene set enrichment analysis

Target genes of the top 20 abundant miRNAs were predicted with miRWalk database V3. Gene ontology (GO) term enrichment analysis was performed with the “ClusterProfiler” R package 4.0 (Wu et al., 2021). Annotation for biological processes was derived for human from org. Hs.e.g.db V 3.21. Significant gene sets were calculated by over representation analysis and adjusting for multiple testing using Benjamini Hochberg (BH). Gene sets with an adjusted p-value <0.05 were considered as significantly enriched. Based on the adjusted p-value derived from the GO enrichment analysis, the 20 top significantly biological processes were visualized with ggplot2 v3.4.2 as a dot plot. Analyses were performed with R version 4.4.0.

### 2.10 Statistics

MiRNA concentration data were analyzed using GraphPad-Prism software (GraphPad Software 10.1.2, United States) by Mann-Whitney test. Level of significance was set at 0.05 and is indicated as ****p < 0.0001.

## 3 Results

Extracellular miRNA can be found either associated with cell-secreted extracellular vesicles (EVs) or bound to proteins. Before analyzing miRNAs using next generation sequencing, we confirmed the presence of EVs in our preparations. EVs were enriched from the conditioned medium of hAM biopsies and preparations analyzed for EV-specific parameters. Beside the particulate SEC elution profile, we measured the protein elution profile in the SEC fractions (as is outlined in Figure 1).

### 3.1 EV biomarkers confirm presence of EVs in hAM secretome

**FIGURE 2:**
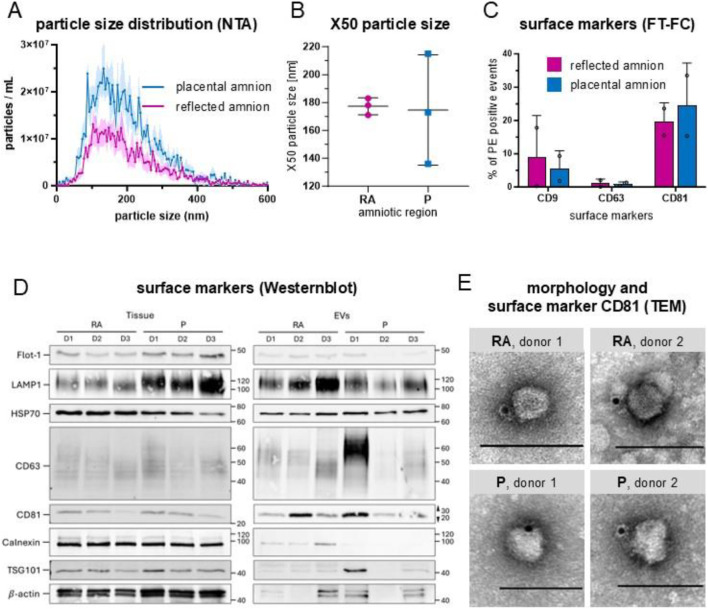
Characterization of hAM-derived extracellular vesicles. **(A)** Particle size distribution by nanoparticle tracking analysis (NTA) showed that most particles were ≤200 nm; n = 3, mean ± SEM. **(B)** Median values (X50) of particle size range from 138 nm to 211 nm after 72 h of incubation (x̄ = 177 in RA and x̄ = 174 in P), n = 3. The majority of particles were ≤200 nm. X50 values are indicated ±SD. **(C)** Fluorescence-triggered flow cytometry analysis (FT-FC) showed that 20%–35% of the EVs were positive for CD81 (x̄ = 19.55 in RA, x̄ = 24.40 in P). The expression of CD9 was less pronounced n = 2. **(D)** Western Blot analysis showed that CD63 and CD81 were more abundant in EVs than tissue, n = 3. **(E)** TEM confirmed EV morphologies and CD81 positive particles (immuno-gold particles (black dots)) in EV preparations from RA and P. scale bar = 100 nm. Abbreviations: human amniotic membrane (hAM), extracellular vesicles (EV), placental amnion (P), reflected amnion (RA), flotillin-1 (Flot-1), lysosomal associated membrane protein 1 (LAMP1), heat shock protein 70 (HSC70), tumor susceptibility gene 101 (TSG101), cluster of differentiation (CD), transmission electron microscopy (TEM).

After SEC enrichment of placental and reflected hAM conditioned media, we measured the protein concentration in each SEC fraction to separate the fractions enriched in EVs from those low in particles and enriched in proteins (Supplementary Figure S1). Then, we tested for EV presence. NTA size distribution analysis revealed that most particles were sized ≤200 nm, indicating a predominant enrichment of small EVs (sEVs) from both hAM regions (Figure 2A)., The median size distribution of around 180 nm indicates predominantly small-sized EVs, with P showing higher variation (Figure 2B). FT-FC showed that 20%–35% of the lipid dye CMG^+^ events were positive for CD81 (Figure 2C), confirming the presence of EV characteristic proteins present on detected particles. These data also suggest that the sEV population is heterogeneous with some vesicles expressing CD81 and others not. The amount of CD9 positive sEVs, another member of the tetraspanin protein family, was markedly lower (Figure 2C), while CD63 positive events were almost absent (Figure 2C).

We also performed Western blot analysis on isolated sEVs using reflected and placental amnion tissue as controls examining transmembrane (CD63, CD81, LAMP1), cytosolic (TSG101, FLOT1, HSP70, β-actin), and intracellular compartment proteins (calnexin) (Figure 2D; Supplementary Figure S2A–C). CD63 and CD81 were more abundant in EVs than tissue, while LAMP1 showed inverse trends between RA and P regions. Flot-1 and TSG101 were higher in tissue, and HSP70 was more abundant in RA tissue, with slight variability in EVs. β-actin was significantly lower in EVs. Calnexin was highly expressed in tissue but nearly absent in EVs, confirming EV purity. While the immunophenotype and size of enriched sEVs are indicative of contributions from endosomal biogenesis routes, the observed inter-donor variability and regional differences in placental topology suggests a heterogeneous population, likely comprising both sEVs from endosomal and ectosomal origin.

EV morphology and identity were assessed by TEM combined with CD81 immunogold labelling. Particles exhibiting the typical nearly spherical morphology and negative contrast commonly associated with EVs in drop-on-grid stainings were observed. While cup-shaped structures were not detected, a subset of these negatively contrasted particles showed CD81 positivity (Figure 2E), confirming their identity as EVs. Importantly, only CD81-positive particles are shown in Figure 2E to demonstrate the presence of EVs in the conditioned medium of RA and P, without further morphological classification or quantification.

Taken together, these data confirm EV presence in the conditioned medium of RA and P. Based on these results, miRNA analysis was carried out separately for the “EV fraction” and the “protein fraction” in correlation to tissue miRNA preparations.

### 3.2 Classification of small RNAs identified diverse RNA species in hAM-derived samples

**FIGURE 3:**
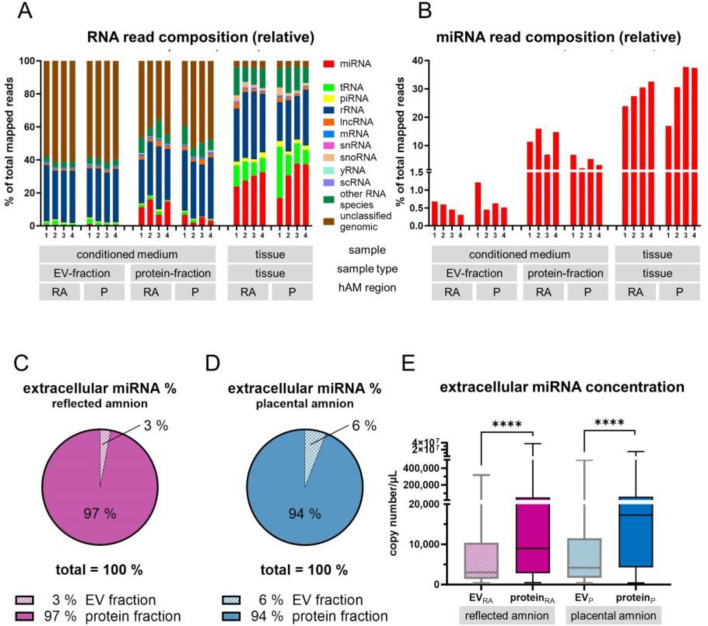
Classification of small RNAs. **(A)** Small RNAs were determined by Next Generation Sequencing in the conditioned medium (EV-fraction and protein-fraction) and in tissue of the placental and reflected region of the hAM. **(B)** The highest percentage of miRNA reads relative to all mapped reads specific to that sample type was found in tissue samples (15%–40%). 2%–15% were found protein-bound in the conditioned medium, and 0.3%–1.2% EV-associated in the conditioned medium. **(C,D)** From the total extracellular miRNA reads, 3% were EV-associated and 97% were protein-bound from samples of the reflected region, and 6% were EV-associated and 94% were protein-bound from samples of the placental region. **(E)** In the conditioned medium, comparison of miRNA concentrations showed significantly higher concentrations in the protein fraction of the reflected and the placental region compared to the respective EV-fractions. ****p < 0.0001.

Next, we determined the small RNA profile of hAM tissue, the EV-fraction and protein fraction of the conditioned medium of the placental and reflected region. We found that the main RNA repertoire included microRNA (miRNA), transfer RNA (tRNA), pivi-interacting RNA (piRNA), ribosomal RNA (rRNA), long non-coding RNA (IncRNA), messenger RNA (mRNA), small nuclear RNA (snRNA), small nucleolar RNA (snoRNA), yRNA, and small cytoplasmic RNA (scRNA) (Figure 3A). Tissue samples exhibited the highest percentage of miRNA reads relative to the total mapped reads specific to that sample type (15%–40%, mean 28.61 in the reflected region and 30.67 in the placental region, Figure 3B). In the conditioned medium, we found 2%–15% miRNA in protein-bound (mean 12.25 in the reflected region and 4.31 in the placental region) and 0.3%–1.2% in EV-associated small RNAs (mean 0.51 in the reflected region and 0.70 in the placental region, Figure 3B). From the total extracellular miRNA pool, 3% were EV-associated and 97% were protein-bound from samples of the reflected region (Figure 3C), and 6% were EV-associated and 94% were protein-bound from samples of the placental region (Figure 3D). Comparison of miRNA concentrations of the conditioned medium showed significantly higher concentrations in the protein fraction of the reflected and the placental region compared to the respective EV-fraction (Figure 3E). The total number of identified miRNAs was 1,589 in all samples (data not shown). To analyze overlaps between the top 20 abundant miRNAs of each conditioned medium group (Supplementary Table S1), we created a Venn diagram (Supplementary Figure S3). A subset of 11 miRNAs was consistently detected in both extracellular fractions (EV and Protein) for both regions (RA and P). EV-fraction of RA and EV-fraction of P share 5 miRNAs, while protein-fraction of RA and protein-fraction of P share 4 miRNAs. Only 1 shared miRNA was found when analyzing EV-fraction of RA ∩ EV-fraction of P ∩ protein-fraction of RA or EV-fraction of P ∩ protein-fraction of RA ∩ protein-fraction of P or EV-fraction of RA ∩ EV-fraction of P ∩ protein-fraction of P (∩ = symbol indicating intersection). The number of unique miRNAs was 2 for the EV-fraction of RA, 1 for the EV-fraction of P, 3 for the protein-fraction of RA, and 3 for the protein-fraction of P (Supplementary Figure S3).

### 3.3 Unsupervised cluster analysis identified distinct miRNA profiles in tissue, EV fraction, and protein fraction

**FIGURE 4:**
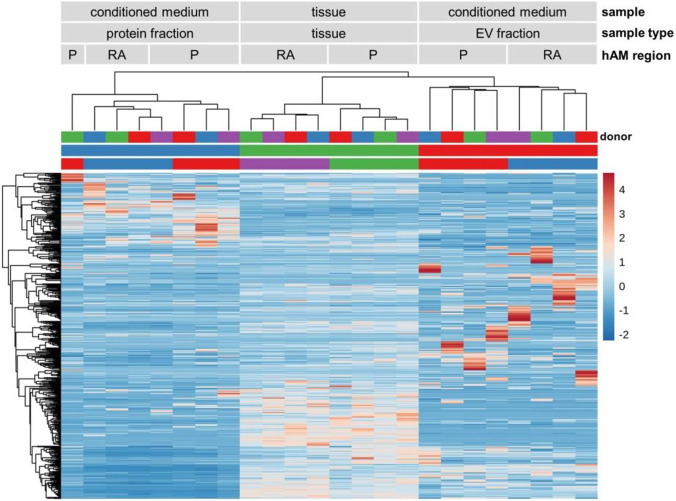
miRNA heat map and dendrogram. Unsupervised hierarchical clustering of miRNA expression. Three main clusters were identified, based on the sample type (protein fraction, tissue, and EV fraction). High expression of specific miRNAs was found in each cluster. Subcluster formation corresponding to the amniotic regions (placental and reflected amnion) were also observed. n = 4. Abbreviations: extracellular vesicle (EV), placental amnion (P), reflected amnion (RA).

To detect miRNA expression patterns and to evaluate sample type and hAM location similarities, we performed unsupervised hierarchical clustering and visualized results in a heat map together with a dendrogram. In the heat map, 504 miRNAs are shown to identify three specific main clusters (Figure 4). Interestingly, these clusters matched according to the sample type, i.e., protein-fraction, tissue, EV fraction. Different patterns of highly expressed miRNA were observed in the protein fraction, in the tissue, and the EV fraction. In addition, in each cluster, we found subcluster formation according to the region of the amniotic membrane, the reflected region and the placental region (Figure 4). Also, the dendrogram confirmed that the protein-bound miRNAs and EV-associated miRNAs are substantially different from each other. In addition, the EV associated miRNA profile better reflects the miRNA content found in tissue compared to protein-bound miRNA expression profiles (Figure 4).

### 3.4 Differential miRNA expression across groups and amniotic regions

**FIGURE 5:**
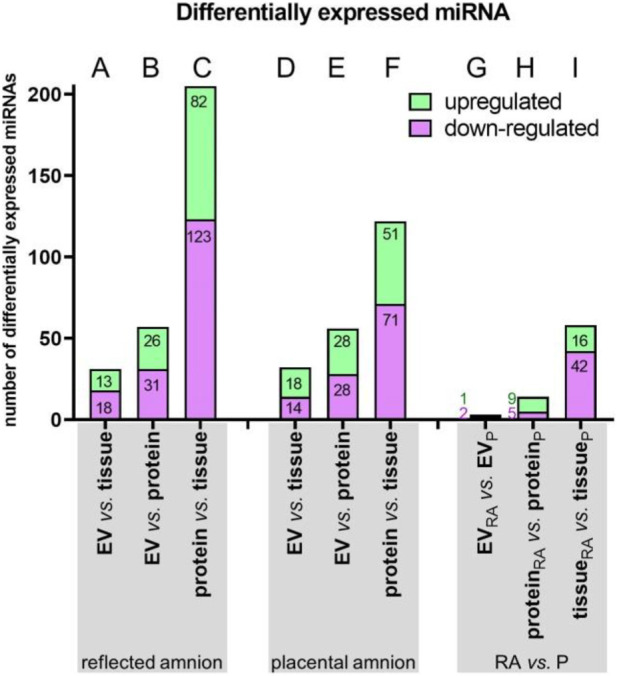
Summary of differential miRNA expression analysis. Within the groups of reflected **(A–C)** and placental amnion **(D–F),** miRNA expressions of the sample fractions (EV-associated, protein-bound and tissue) were compared. Additionally, miRNA expressions of the amniotic regions reflected (RA) versus placental amnion (RA) were compared **(G–I)**. EV-associated miRNAs, protein-bound miRNAs or miRNAs of reflected and placental amnion showed differentially up- or downregulated miRNAs. Numbers of significantly up/downregulated miRNAs with a false discorvery rate (FDR) < 0.05 are represented in a bar chart. Upregulated = logFC >0; down-regulated = logFC <0.

Next, to identify miRNAs that are over- or underexpressed compared to other groups, we used differential expression analysis (Figure 5). We found a number of significantly upregulated (green bar) and downregulated (purple bar) miRNAs when comparing different fractions of RA (Figures 5A–C; Supplementary Table S2A–C) and P (Figures 5D–F; Supplementary Table S2D–F). In addition, comparison between the reflected and placental region showed a number of differentially expressed miRNAs between these two amniotic regions (Figures 5G–I; Supplementary Table S2G–I). We found the most differentially regulated miRNAs when we compared protein-bound and tissue miRNAs in the reflected amnion (82 up-regulated/123 down-regulated) and the placental amnion (51 up-regulated/71 down-regulated). The lowest number of differentially regulated miRNAs was found when comparing EV-associated miRNAs between reflected and placental region (1 up-regulated/2 down-regulated).

### 3.5 Gene ontology enrichment analysis reveals distinct functional pathways for miRNAs across sample types and regions

**FIGURE 6:**
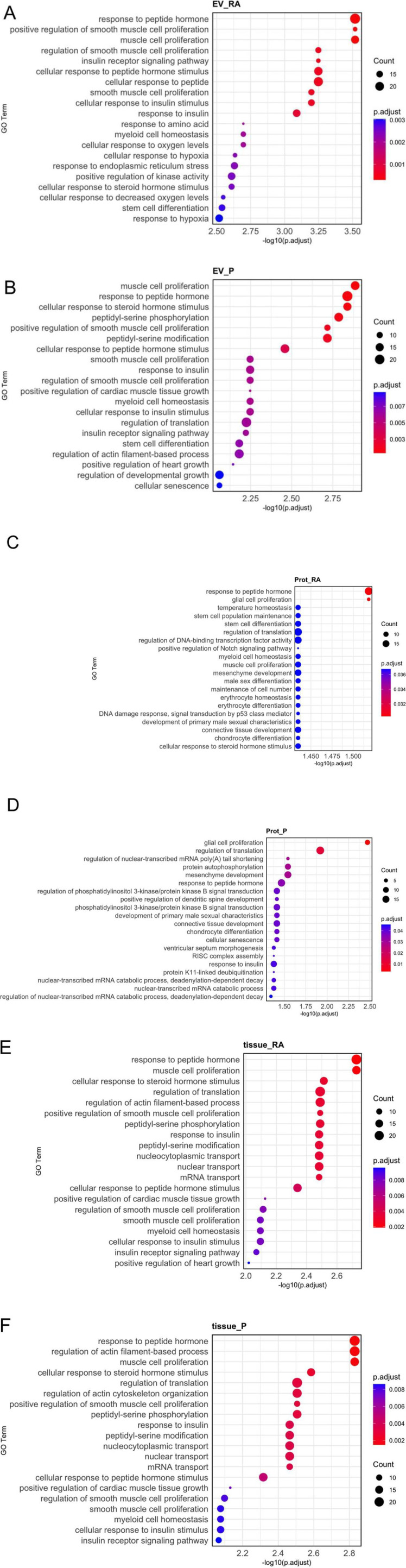
Gene ontology (GO) enrichment analysis of biological processes. The 20 most abundant miRNAs from each fraction (EV-associated, protein-bound, or tissue) and amniotic region (reflected or placental) were analyzed. EV-associated miRNAs **(A,B)** and tissue miRNAs **(E,F)** were related to muscle and smooth muscle development pathways. Protein-bound miRNAs **(C,D)** were prominently linked to pathways connected to glial cells, connective tissue, and chondrogenesis. Adjusted p-value <0.05 were considered as significantly enriched. The x-axis represents the significance of each pathway as [-log10 (adjusted p-value)]. The size of each circle represents the number of genes associated with the GO term and the color indicates the adjusted p-value.

To evaluate possible biological functions of miRNAs identified in our study, we performed gene ontology enrichment analysis with the 20 most abundant miRNAs per fraction. On one hand, EV-associated and protein-bound miRNAs are of interest as released miRNAs can be taken up by acceptor cells. On the other hand, intracellular miRNAs are also of interest as they most likely impact the amniotic gene regulation/expression (Figure 6; Supplementary Table S3). Among the 20 most significantly enriched pathways, analyses showed predominantly ubiquitous cellular pathways, such as cellular senescence, response to insulin, cell differentiation or response to hypoxia. In addition, EV-associated miRNAs (Figures 6A,B) and tissue miRNAs (Figures 6E,F) are involved in various muscle and smooth-muscle cell related pathways, whereas protein-bound miRNAs (Figures 6C,D) are more involved in pathways related to glial cells, connective tissue, and chondrogenesis.

### 3.6 Correlation analysis revealed distinct secreted EV-associated/protein-bound miRNAs

**FIGURE 7:**
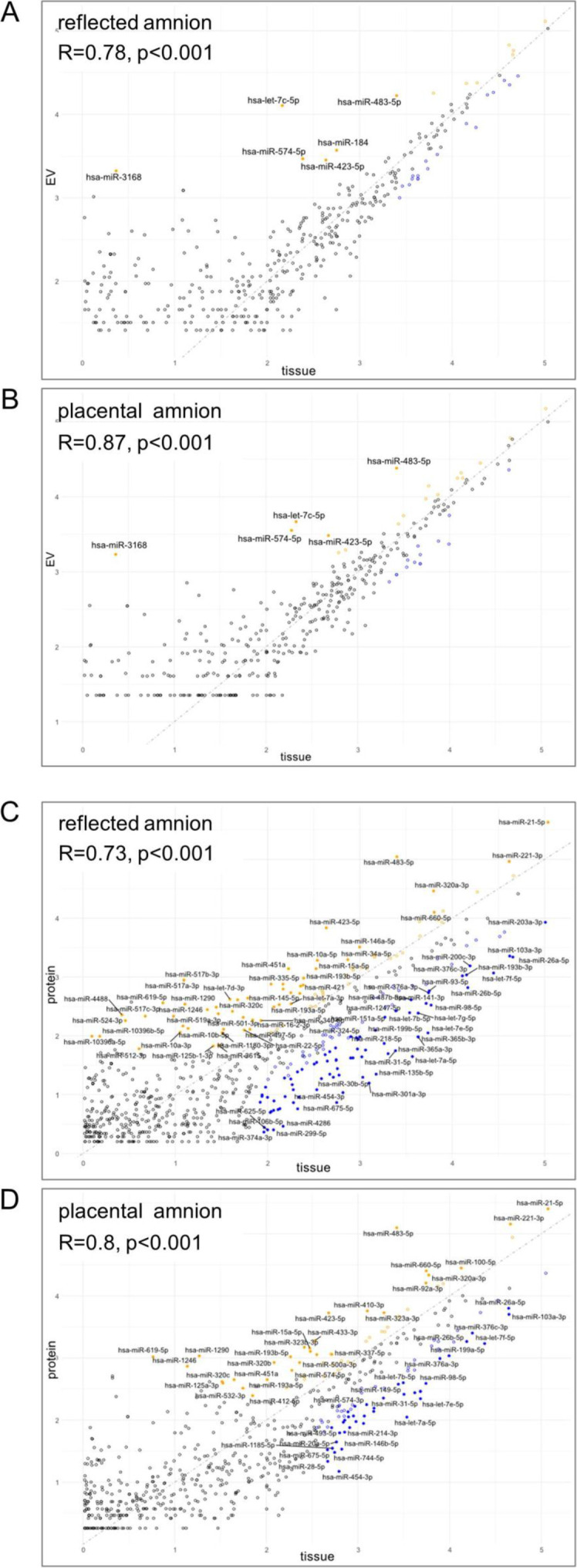
Correlation of tissue miRNAs and extracellular expression. Spearman correlation of **(A)** EV-associated and tissue level of miRNAs of reflected amnion, **(B)** EV-associated and tissue level of miRNAs of placental amnion, **(C)** protein-bound and tissue level of miRNAs of reflected amnion, and **(D)** protein-bound and tissue level of miRNAs of placental amnion. miRNAs with an FDR <0.05 and an absolute logFC >2 are highlighted and labeled (yellow = extracellular, blue = tissue). The grey line represents a perfect correlation with a slope of 1. For the list of miRNAs see Supplementary Table S4.

Finally, we analyzed if the extracellular abundance of miRNAs correlates with the miRNA abundance in the tissue to identify enrichment of EV-associated or protein-bound miRNAs. (Figure 7). In the EV-associated fraction, we found 6 miRNAs in RA (Figure 7A; Supplementary Table S4A) and 5 miRNAs in P (Figure 7B; Supplementary Table S4B), which may specifically be loaded into EVs in these regions. Correlation of the protein-bound fraction with the tissue fraction showed 37 miRNAs in the RA (Figure 7C; Supplementary Table S4C) and 25 miRNAs in the P region (Figure 7D; Supplementary Table S4D), which may specifically be released in the extracellular space but not packed into EVs.

## 4 Discussion

Although the hAM plays a pivotal role during pregnancy and birth, there are still many open questions regarding the regulation of various processes in the hAM. For example, to this day, the interactions between the hAM, the amniotic fluid and the fetus during pregnancy are not well understood and partially unknown. Biological and secretory functions of amniotic cells affect the biochemical composition of the amniotic fluid (Kumar et al., 2006) and could play a role for the development of the fetus (Lemke et al., 2017; Shamsnajafabadi and Soheili, 2022). Moreover, processes in the hAM might also contribute to the onset of labor (Menon and Fortunato, 2004; Menon, 2019). Given the substantial bioactivity of the tissue, the use of hAM, its cells, or its conditioned medium has been suggested for a number of tissue regeneration procedures, due to their high regenerative potential (Silini et al., 2020; Janev et al., 2023). Since miRNAs are known to influence physiological/pathophysiological signal transduction by modulating the availability of signaling components, we aimed at assessing a comprehensive miRNA profile of the hAM. We investigated the abundance of miRNAs in hAM tissue and hAM conditioned medium. Among the miRNAs found in the conditioned medium, we further differentiated between EV-associated (vesicular) and protein-bound miRNAs (non-vesicular).

It is assumed that miRNAs are secreted to be taken up by surrounding cells (Makarova et al., 2016). One possible application of the hAM or its cells for therapeutic applications is the production of secretome preparations to make use of released factors. Therefore, one aim of our study was to investigate the *ex vivo* miRNA secretion behavior of the hAM. In general, we found that miRNA levels in the tissue were much higher compared to the fractions in conditioned medium (EV-associated and protein-bound). In the conditioned medium, we found higher levels of protein-bound miRNAs compared to EV-associated miRNAs. This is in line with studies of human blood plasma (Arroyo et al., 2011) and conditioned medium from MCF7 cells (Turchinovich et al., 2011). In these studies, most of the extracellular miRNAs were of non-vesicular origin and only a minority of miRNAs was associated predominantly with vesicles. A possible explanation has already been suggested by others (Geekiyanage et al., 2020). It is likely that under normal conditions, cells release predominantly protein-bound miRNAs whereas under pathological conditions, levels of EV-associated miRNAs increase (Geekiyanage et al., 2020). If this is the case, this needs to be taken into consideration for the production of therapeutic EVs of the hAM or its cells, including strategies such as inflammatory/hypoxic priming into the preparation protocol. This could pave the way for more specifically tailored therapy approaches that could be designed through specific stimulations. Alternatively, a maintained cell viability within the grafting material may allow an adapted secretory response to the local pathology upon transplantation.

The fact that some miRNAs are released via EVs and some bound to proteins suggests that the secretion of miRNAs in the hAM happens in a specific manner, which was also demonstrated by hierarchical cluster analysis. The observed miRNA expression patterns reveal three distinct signatures across sample types, suggesting that specific miRNAs may play a role in the cellular processes of the hAM. This also means that the choice of sample type could have an impact on the therapeutic effect of the hAM. Interestingly, when analyzing similarities, protein-bound miRNAs were substantially different compared to EV-associated and tissue miRNAs, and EV-associated miRNAs were more similar to tissue miRNAs. The former could be explained by the assumption that protein-bound extracellular miRNAs may have signaling functions that differ from those of vesicle-associated miRNAs. This difference could provide hAM cells with a flexible range of signaling options, again implying that the type of secretome preparation for therapeutic applications may have an influence on the outcome. In this context, Castoldi and colleagues observed that serum vesicular- and non-vesicular miRNAs were modulated differently in animals recovering from partial hepatectomy (Castoldi et al., 2016), leading to the speculation that this may help coordinate responses in both close and distant cells during the various phases of liver regeneration (Castoldi et al., 2016). Regarding non-vesicular miRNAs, it has been discussed that these could be mostly by-products of dead cells (Turchinovich et al., 2011). However, the fact that protein-bound miRNAs of the hAM show a specific pattern that differs from that of the tissue, indicates that these miRNAs are not merely passively released from dead cells of the hAM.

Beside cluster trees according to the sample type, we found two sub-trees according to the two sub-regions of the amniotic membrane, the reflected and the placental region, in each sample type. This is in accordance with previous studies, where we and others have shown that cells of the two amniotic sub-regions, reflected and placental, have different cellular and metabolic properties (Weidinger et al., 2021). In the light of tissue regeneration processes, this could be another important indicator to utilize the sub-regions of the hAM for different therapeutic purposes. Regarding metabolism, it has been shown that mitochondrial respiratory chain activity and intracellular adenosine triphosphate levels affect the exporting process of miRNAs (Wang et al., 2010; Chatterjee et al., 2021). Of note, it has been shown that mitochondrial activity and ATP levels are different in the placental and reflected region of the hAM (Weidinger and Banerjee, 2020), which may influence the exportation of miRNAs.

To further identify most suitable sample types as the source of specific miRNAs for tissue regeneration, we also performed differential analysis of miRNA abundance. Regarding the amniotic regions, we found 75 differentially expressed miRNAs between the reflected and the placental amnion in the three sample types. For example, miR-143 and miR-145 were upregulated in the placental amnion and downregulated in the reflected amnion. This is in line with a previous study, where the authors pointed to a possible effect on parturition (Kim et al., 2011). Accordingly, miR-143 and miR-145 have recently been suggested to play an important role in the breakdown of the cervical epithelial barrier by targeting cell adhesion and anti-apoptotic genes (Anton et al., 2017). Furthermore, it has been shown that miR-143-3p is involved in vascular adaptation during pregnancy (Liu et al., 2023). This miRNA plays a key role in uterine spiral artery remodeling by inducing vascular smooth muscle cell apoptosis to promote the development of extravillous trophoblast differentiated cells (Liu et al., 2023). During placental development, extravillous trophoblasts remodel about 100–150 uterine spiral arteries, transforming them from high-resistance, low-flow vessels to high-flow, low-resistance channels (Lyall, 2005). These processes involve placenta and changes are critical for proper placentation and fetal development (Burton et al., 2009). In a regenerative context, it has been shown that cortisol-induced miR-143/145 expression in monocytes suppresses M1 macrophages and supports polarization to M2 macrophages, a state that has been associated with resolving inflammation and support of tissue regeneration (Sharma et al., 2024). This is just one example where miRNA, which was probably originally produced for the processes before and during birth, could be used for therapeutic purposes.

We also conducted a gene ontology enrichment analysis to explore the potential biological functions of the miRNAs identified in our study. Interestingly, among the top 20 pathways, beside pathways linked to broader cellular processes, we identified several pathways significantly associated with muscle, smooth muscle and cardiac muscle proliferation as target tissues of EV-associated miRNA and tissue miRNA. Throughout embryonic development, muscle formation is a continuous process. By 31–33 weeks of gestation, human prenatal muscle growth involves an increase in type I muscle fibers, and skeletal muscle cells likely become multinucleated (Romero et al., 2013). Three key factors appear to influence muscle maturation including cell fusion, alignment of fusing cells, and filament synthesis. Disruptions in any of these factors may result in muscle fiber abnormalities early in muscle development. Given the importance of these developmental processes, it is not surprising that miRNAs associated to muscle cell development are highly present in the EVs and tissue of hAM. These miRNAs likely play a regulatory role in muscle cell development and maturation during fetal development.

However, these data are not only interesting regarding the process of fetus development and pregnancy, also possible conclusions for tissue regeneration could be drawn. Enhancing cardiac muscle proliferation could transform treatments for heart failure, a leading cause of morbidity and mortality worldwide. Recently, it has been shown that human amniotic fluid-derived stem cell EVs promote cardiac repair and stimulate regeneration by modulating endogenous mechanisms through paracrine signaling after a single intra-myocardial injection (Balbi et al., 2019). Integrating miRNA therapy with stem cell secretome strategies could further optimize cardiac repair and regeneration interventions.

When analyzing protein-bound miRNAs, we found pathways linked to connective tissue development, chondrocyte differentiation, and glial cell proliferation among the top 20 biological functions. Gliogenesis is an important mechanism to maintain and regulate brain function in the late phase of development but also after birth (Arai and Lo, 2017). Regarding tissue regeneration, studies have also shown that the number of glial cells increases significantly in response to injury (Ardaya et al., 2025), supporting tissue repair (Stassart et al., 2013).

It has been shown that the cell-free transcriptome (Tarca et al., 2020) and proteome (Bhatti et al., 2022) of the amniotic fluid change with gestational age, supporting the speculation that the amniotic fluid could be a means of transportation to support the development of the fetus (Tong et al., 2009). In this context, miRNAs of the hAM released to the extracellular space are of special interest because these miRNAs could also play a role for tissue regeneration. We therefore analyzed if cells in hAM tissue secreted EV-associated and protein-bound miRNAs selectively. For this purpose, we performed correlation of extracellular abundance of miRNAs and tissue abundance to identify miRNAs which may specifically be loaded into EVs or bound to proteins. Interestingly, among those found, many were related to neuroprotection/regeneration. In line with this, Castelli et al. found similar results when analyzing the exosomal fraction of human amniotic fluid samples (Castelli et al., 2021).

Accordingly, we identified several selectively released miRNAs in the EV fraction in both RA and P that are known to play roles in brain and peripheral nerve function and regeneration. These include hsa-let-7c-5p, hsa-miR-574-5p, hsa-miR-184, hsa-miR-483-5p, and hsa-miR-423-5p. MiRNA hsa-let-7c-5p has been shown to support nerve regeneration by inhibiting neuroinflammation, reducing microglial activation (Lv et al., 2018), and promoting myelination through Krox20 expression (Gökbuget et al., 2015). In a mouse model, overexpression of miR-574-5p in the hippocampus reduced β-site amyloid precursor protein cleaving enzyme 1 (BACE1 levels), restored synaptic function, and improved memory impaired by pollution exposure (Ku et al., 2017). Following ischemic stroke in rats, it has been demonstrated that miR-184 expression changes in the brain (Yang et al., 2021). This study also revealed that overexpressing miR-184 increased cell viability and reduced apoptosis in SH-SY5Y cells subjected to oxygen-glucose deprivation and reoxygenation *in vitro* (Yang et al., 2021). Similarly, miR-483-5p has been shown to regulate neuronal metabolism and help mitigate neurological injury following cardiac arrest by decreasing the expression of the pro-apoptotic protein Bax, thereby inhibiting the release of cytochrome c from mitochondria (Zhang et al., 2023). Finally, in a rat model of spinal cord injury, miR-423-5p modulated inflammation by targeting the NLRP3 inflammasome, preventing M1 microglial polarization, and promoting recovery (Cheng et al., 2021).

In addition, we also found several miRNAs in the protein fraction, such as hsa-miR-21-5p, hsa-miR-340, hsa-miR-221-3p, and miR-210 that have been shown to be implicated in nerve regeneration. Hsa-miR-21-5p and hsa-miR-221-3p have been shown to inhibit apoptosis and enhance cell viability (Zhou et al., 2015). Specifically, miR-21-5p promotes axon growth in dorsal root ganglion (DRG) cultures (Ning et al., 2020; Kar et al., 2021) and inhibits nerve inflammation and neuropathic pain (Zhong et al., 2019). MiR-21 further supports Schwann cell proliferation and axon regeneration by potentially targeting TGFβI, TIMP3, EPHA4, and caspases-3 and -9 (Ning et al., 2020). Additionally, the hsa-miR-221/222 cluster is reported to regulate Schwann cell phenotype, proliferation, and migration *in vivo* (Yu et al., 2012). Overexpression of miR-210 may support sensory axon regeneration and apoptosis inhibition via ephrin-A3 (Hu et al., 2016). In addition, inhibition of endogenous miR-210 in DRG neurons impairs axon regeneration both *in vitro* and *in vivo* (Hu et al., 2016). MiR-340 influences fibrinolytic activity and enhances Schwann cell migration by modulating tissue plasminogen activator secretion (Li et al., 2017). In a rat model of chronic constriction injury, miR-340 overexpression also reduced inflammation as well as the expression levels of cyclooxygenase 2, interleukin-1β, tumor necrosis factor α and interleukin-6. Some of the neuroprotective miRNAs that were selectively released, such as hsa-miR-21-5p, hsa-miR-221-3p and hsa-miR-483-5p are also found among the top-most abundant miRNAs. Taken together, these data suggest neuroprotective effects of the conditioned medium of the hAM.

## 5 Conclusion

The analysis of miRNAs of the hAM opens an entire new world for the understanding of this remarkable tissue. Many properties of the hAM have been thoroughly investigated and described, yet when it comes to the situation *in utero*, these properties do not quite fall into place. It is not clear, if miRNAs, involved in smooth muscle, muscle or cardiac muscle functions, are required by the fetus, the mother, or both. In addition, the distinct packaging of miRNAs seems to play an important role for the subsequent molecular function. Consequently, this study raised several new questions that will require further investigation. The analysis of the miRNA profile was a first step. However, miRNAs are known to act in a complex manner and different ways to fine-tune levels of specific proteins that are involved in various signaling pathways. Thus, further studies are necessary to investigate the actual effects of miRNAs. In addition, the hAM is formed very early during gestation and expires with childbirth. This means, our analysis is merely a snapshot in time. It is very likely that different miRNA profiles are expressed and secreted at different time points.

Further studies are also necessary to examine if amniotic miRNAs are active and which functions - activation or repression - are executed by a single miRNA or cluster of miRNAs under certain circumstances. This will not only help to better understand tissue regeneration processes but also provide more information about gestation, the onset of labor and the hAM in general, a tissue every single human life was nurtured and protected with before being born.

## Data Availability

Data of this study have been deposited in the NCBI Gene Expression Omnibus (GEO) under the accession number GSE306157.

## Abbreviations

hAM, human amniotic membrane; RA, reflected amnion; P, placental amnion, EV, extracellular vesicles.
