# Supplementary material for: Distinct miRNA profiles in human amniotic tissue and its vesicular and non-vesicular secretome
Source: Front Cell Dev Biol. 2025 Oct 29;13:1692501. doi: 10.3389/fcell.2025.1692501 (PMC12605191; doi:10.3389/fcell.2025.1692501)
Supplement: Supplementary file 5 [file DataSheet1.docx]

Supplementary Material

# Supplementary Figures

##
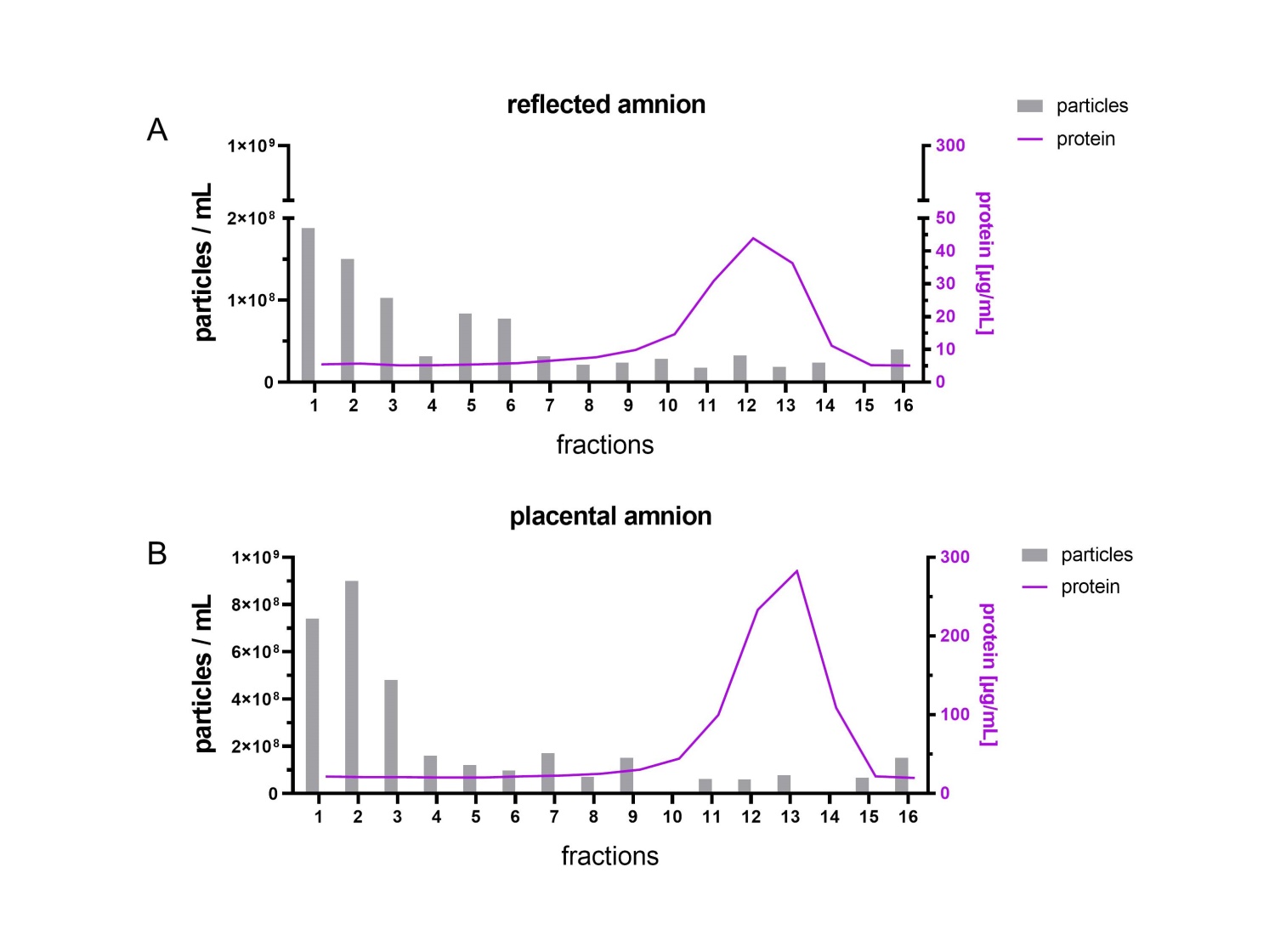
Supplementary Figure 1

**Supplementary Figure 1.** **EV *vs.* protein fraction in conditioned medium.** Particles were detected with nanoparticle tracking analysis and protein concentration was measured by bicinchoninic acid protein assay in 1 mL conditioned medium. Both **(A)** reflected and **(B)** placental amnion conditioned medium showed increased protein concentration in fractions 9 to 15.

## Supplementary Figure 2


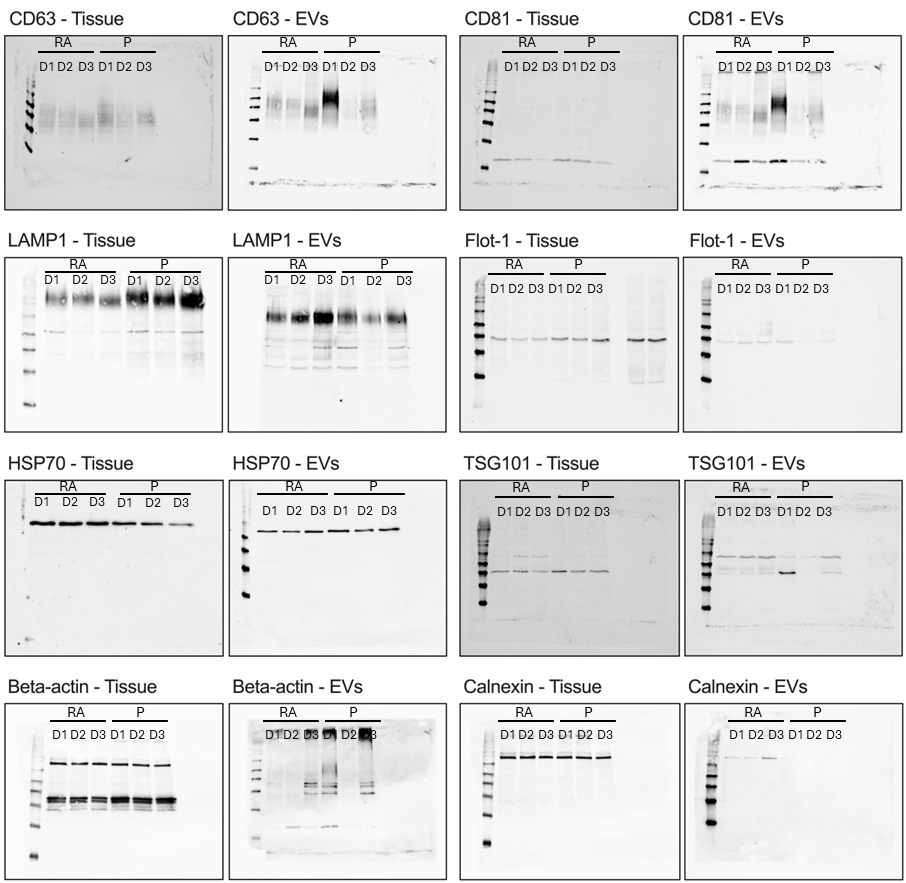


**Supplementary Figure 2A.** **Native western blot.** Native western blot of the characterization of protein content of EVs isolated from reflected (RA) and placental (P) amnion after incubation for 72 h. Transmembrane (CD63, CD81, and LAMP1) and cytosolic proteins (TSG101, Flot1, HSC70, and calnexin) were analyzed. hAM tissue was used as a control. Biological replicates n = 3. Reflected amnion (RA), placental amnion (P), extracellular vesicles (EVs), cluster of differentiation (CD), lysosomal associated membrane protein 1 (LAMP1), flotillin-1 (Flot-1), heat shock protein 70 (HSC70), tumor susceptibility gene 101 (TSG101).

**Supplementary Figure 2B.** **Western blot relative protein expression tissue.** Procedure: Normalisation of individual BG-corrected values to ctrl (b-actin as loading control) and calculation of the expression fold difference P to RA. Paired Student`s t-test, p<0.05 assumed statistically significant. Reflected amnion (RA), placental amnion (P), cluster of differentiation (CD), lysosomal associated membrane protein 1 (LAMP1), flotillin-1 (Flot-1), heat shock protein 70 (HSC70), tumor susceptibility gene 101 (TSG101).

**Supplementary Figure 2C.** **Western blot relative protein expression EVs.** Procedure: No normalisation of individual BG-corrected values to any loading ctrl (equal loading assumed based on Coomassie blue membrane staining). Calculation of the expression fold difference P to RA. Paired Student`s t-test, p<0.05 assumed statistically significant. Reflected amnion (RA), placental amnion (P), extracellular vesicles (EVs), cluster of differentiation (CD), lysosomal associated membrane protein 1 (LAMP1), flotillin-1 (Flot-1), heat shock protein 70 (HSC70), tumor susceptibility gene 101 (TSG101).

##
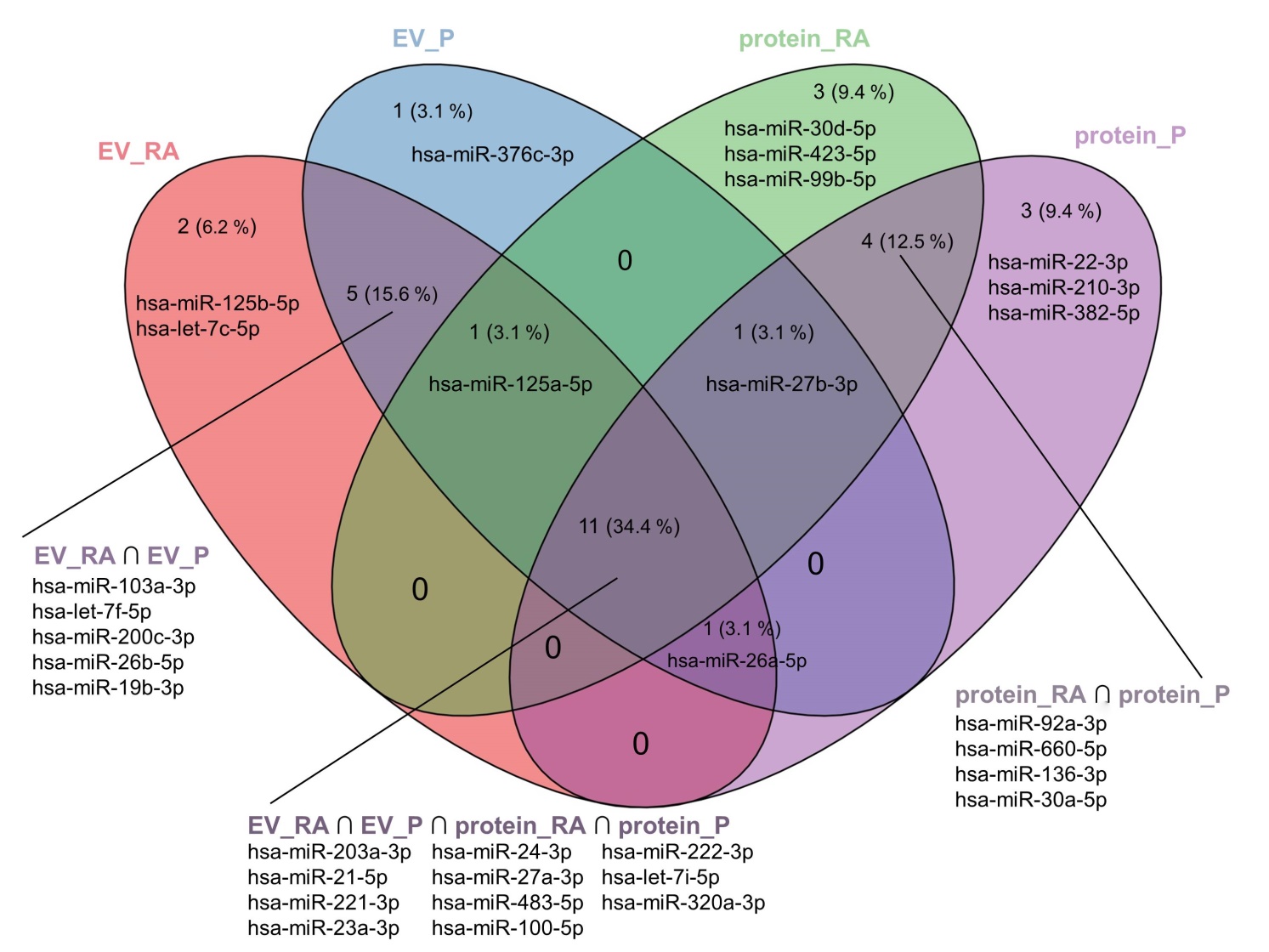
Supplementary Figure 3

**Supplementary Figure 3.** **Venn diagram of Top 20 most abundant miRNAs.** Venn diagram shows unique miRNAs and overlap between the top 20 miRNAs in each sample type and amniotic region. RA, reflected amnion; P, placental amnion; EV, extracellular vesicles. ∩ = symbol indicating intersection.
